# “A thought I can talk to”: dialogical self-regulation through spontaneous inner voice personification in a gifted adolescent

**DOI:** 10.3389/fpsyg.2026.1815412

**Published:** 2026-05-19

**Authors:** Cora Zeng

**Affiliations:** Independent Researcher, Chengdu, China

**Keywords:** autoethnography, dialogical self, giftedness, imaginary companion, inner speech, inner voice personification, overexcitability

## Abstract

This paper introduces Dialogical Inner Voice Personification (DIVP): a formless, persistent inner dialogue partner that emerges spontaneously in childhood, operates through linguistic turn-taking, and is experienced as part of the self. Drawing on eight autoethnographic interview sessions conducted with an AI research assistant and corroborated by parental accounts, the author — a 14-year-old gifted bilingual student (CAT4 Non-verbal SAS 131, 98th percentile; Spatial SAS 139, 99th percentile) — traces DIVP’s phenomenology and developmental trajectory from early childhood to adolescence, situating it within five intersecting literatures: inner speech development, imaginary companions, Dialogical Self Theory, giftedness and overexcitability, and non-pathological voice-hearing. Each accounts for a component in isolation; none captures the configuration that arises when all five converge. The paper maps this convergence point, proposes a dual-overexcitability mechanism (intellectual × imaginational) as a candidate explanation, and examines the clinical risk of misdiagnosis in gifted children reporting such experiences.

## 1 Introduction

When asked to describe my inner voice, I typically respond: “It is a thought in my mind that I can talk to.” Thoughts, in the common assumption, do not talk back. Mine does — in specific words, with its own perspective, on topics I have not consciously chosen. It has been doing this since I was about seven, maybe earlier. I am now 14.

For most of my life I assumed everyone’s thinking worked this way. Only when I tried to explain it and observed others’ reactions did I realize it might not be standard. That realization brought a wave of anxiety I now recognize as common among gifted children: the fear that one’s inner experience signals mental illness—what Webb et al. (2005) call a “quiet crisis.”

This paper grew out of wanting to understand what I had been experiencing. The research question is simple—*What is this phenomenon?*—but answering it required drawing from five areas of psychology, none of which captures it alone.

My inner voice is not an imaginary companion (Taylor, 1999): it satisfies none of Taylor’s seven identity criteria (stable name, visual appearance, personality, gender, emotional life, narrative persistence, understood fictionality). It is not a hallucination: I have never mistaken it for an external voice. It is not a tulpa (Veissière, 2016): I did not construct it. It is not a therapeutic construct (Schwartz, 1995): no therapist was involved. What remains after these exclusions requires its own term.

I call it Dialogical Inner Voice Personification (DIVP). Each element is chosen to distinguish this experience from adjacent phenomena. *Dialogical* connects to Hermans (2001) Dialogical Self Theory and denotes genuine perspectival exchange, not monological self-talk. *Inner voice* (rather than “inner speech”) implies a felt *speaker*—the phenomenological core of DIVP—while ruling out external attribution (Alderson-Day and Fernyhough, 2015). *Personification* captures the defining paradox: I relate to the voice as a *someone*, yet it possesses no identity features — no name, no appearance, no gender. If it had a stable identity, it would be an imaginary companion; if it lacked personification, it would be ordinary dialogical inner speech. DIVP occupies the space between: *personification without identity*.

The argument is not that DIVP is unprecedented, but that it sits at an uninspected intersection of five well-studied domains: inner speech development, imaginary companions, Dialogical Self Theory, giftedness and overexcitability, and non-pathological voice-hearing. Section 2 reviews each and identifies where DIVP falls through the gaps between them.

Research questions:

1. What is the phenomenology of DIVP?
2. What is its developmental trajectory from early childhood to adolescence?
3. How does it relate to existing categories (imaginary companions, I-positions, inner speech)?
4. What role does cognitive giftedness play in its emergence?

## 2 Literature review

### 2.1 Inner speech development

Vygotsky (1934/1986) established that inner speech originates as outer speech: children talk aloud to solve problems, then gradually internalize the conversation. By adulthood, most compress their inner speech into abbreviated fragments — what Fernyhough (2004) terms the “condensed” form. Critically, Fernyhough identified an intermediate stage: *expanded* inner speech, featuring complete sentences and conversational give-and-take, which adults temporarily revert to under cognitive difficulty.

The implication for giftedness is direct. If cognitive difficulty pulls inner speech back toward its expanded, dialogical form, a child for whom complexity is the baseline — routinely synthesizing across domains, weighing competing interpretations — may never complete the condensation process. The dialogue persists because the cognitive demand that sustains it never lifts.

Approximately 75% of adults endorse some dialogical quality in their thinking (Alderson-Day and Fernyhough, 2015; McCarthy-Jones and Fernyhough, 2011). Internal dialogue is normative; what varies is its degree, structure, and whether it involves genuinely distinguishable perspectives. Grandchamp et al. (2019) mapped this variation along three dimensions: condensation, dialogality, and intentionality. On that map, DIVP occupies an extreme corner: maximally dialogical, minimally condensed, and — at the level of initiation — volitional rather than involuntary.

A disambiguation is necessary here. “Intentional” operates at two levels that must not be conflated. *Macro-level*: dialogue initiation is volitional (Grandchamp et al.’s intentionality dimension), distinguishing DIVP from pathological voice-hearing. *Micro-level*: individual utterances carry a quality of spontaneous emergence — content arrives as though it “appears on its own” rather than being deliberately composed (see Section 4.3). These levels describe different grain sizes of the same phenomenon: macro-level control without micro-level directedness.

Neuroimaging corroborates the dialogical account: dialogic inner speech activates the same mentalizing regions used for representing others’ perspectives (Alderson-Day et al., 2016). Whether expanded dialogical inner speech serves as the cognitive baseline for gifted individuals remains untested (cf. Ren et al., 2016, on executive functioning and inner speech frequency). The present case suggests it may.

### 2.2 Imaginary companions and their limits

Taylor (1999) definitional framework requires an imaginary companion (IC) to carry recognizable identity features: a name, an appearance, emotional reactions, and the child’s understanding that it is make-believe. Approximately two-thirds of children under seven create something fitting this profile. My inner voice satisfies none of these criteria — it scores zero on Taylor’s seven-feature checklist.

Hart and Zellars (2006) described a closer analog: “wise guide” ICs functioning as advisors rather than playmates, representing externalized aspects of the child’s inner world that are gradually reabsorbed. The trajectory maps onto my experience, but even their wise guides retain perceptible qualities. Mine has been formless for as long as I can remember.

A developmental reading makes DIVP less anomalous. Children who create ICs internalize speech earlier and more completely (Davis et al., 2013), and adults who had childhood ICs report more conversational, evaluative inner speech (Fernyhough et al., 2019). This suggests ICs and dialogical inner speech are not separate categories but positions along a single developmental continuum. DIVP sits at the far end: past the point where form dissolves but function persists. The companion is not abandoned; it is absorbed into the cognitive architecture, retaining only its dialogical core.

In summary, DIVP is too formless for the IC framework, too personified for the inner speech literature, and too functional to trigger clinical concern. It occupies the interstitial space between these categories.

### 2.3 Dialogical self theory

Hermans (2001), synthesizing William James and Bakhtin, reconceived the self as a multiplicity of *I-positions* — semi-autonomous perspectives engaged in ongoing negotiation. Hermans (2012) emphasized that individuals differ greatly in how many positions they maintain and how actively those positions interact.

DIVP’s architecture maps closely onto the I-position framework: my inner voice holds a distinguishable perspective, enters into dialogue with my “main” perspective, and remains recognizably mine while being separable. The fit is striking except on one critical point: in every documented case, I-positions emerge through structured therapeutic work or researcher-guided exercises (e.g., Dillon, 2012, on gifted adolescents constructing multi-voiced selves via email journals). Nobody prompted me to create mine.

This spontaneous emergence suggests that some minds naturally organize into multi-positional structures. Theorists have long suspected that Vygotskian inner speech and I-positions are related phenomena (Fernyhough, 1996; Wiley, 2006/2016), and classroom research demonstrates that conversational environments foster multi-voiced thinking (Van der Veen et al., 2018). But these remain cases where the external world provides the template. DIVP represents a case in which the *endpoint* of internalization diverges from the typical trajectory.

A critical clarification: the claim is not that DIVP bypasses Vygotskian internalization. The dialogical format originates from social-linguistic experience — internalized conversations with parents, teachers, and peers. What distinguishes DIVP is not the *origin* (which remains social) but the *developmental endpoint*: sustained cognitive load, generated by intellectual overexcitability, may prevent condensation from completing, preserving expanded dialogue as the default mode. The template is Vygotskian; the persistence is driven by cognitive demand (Sections 5.2 and 5.5).

### 2.4 Giftedness and overexcitability

Dabrowski (1964) identified five channels of heightened neurological responsiveness — psychomotor, sensual, intellectual, imaginational, and emotional — collectively termed overexcitabilities (OE). For DIVP, the critical interaction is between intellectual and imaginational OE operating simultaneously at elevated levels. Intellectual OE provides the material worth deliberating; imaginational OE lends that deliberation the phenomenological texture of encounter rather than computation (Piechowski, 2009).

Wood et al. (2024) found that children scoring ≥140 on standardized tests predominantly showed elevated responses across three or more OE channels, with the intellectual-imaginational-emotional combination appearing most frequently. Silverman (2013) argued the difference is qualitative, not merely quantitative. Combined with gifted children’s adult-level metacognitive abilities (Alexander et al., 1995), the conditions for a persistent inner dialogue partner become legible: cognitive material to sustain a conversation (intellectual OE), imaginative capacity to experience it as happening with a *someone* (imaginational OE), and metacognitive awareness to observe the process^1^.

This pathway has not been empirically tested. The prediction is clear — screening for combined high intellectual and imaginational OE should yield elevated dialogical inner speech scores — but the study has not been done. The present case is an existence proof that the endpoint is real; the pathway remains theoretical.

### 2.5 Non-pathological voice-hearing

Johns and van Os (2001) demonstrated that experiences clinicians associate with psychosis—including hearing voices—exist on a gradient extending into the general population. Van Os et al. (2009) refined this into a three-stage model: subclinical experiences are common, mostly transient, and only become clinical when they persist with distress and functional impairment.

Under this continuum model, DIVP scores zero on all three pathological markers: no distress (it is a source of comfort), full volitional control, and consistent internal attribution. Professional writers report structurally similar experiences—nearly two-thirds hear their characters speak, and ~14% hold back-and-forth conversations with them (Foxwell et al., 2020). The tulpa community demonstrates that deliberately cultivated inner presences are overwhelmingly experienced positively (Veissière, 2016).

The clinical relevance is sharpest for gifted children. Webb et al. (2005) documented systematic misdiagnosis of overexcitability-driven experiences as ADHD, OCD, autism, or mood disorders. My own case illustrates the complementary risk: periods of genuine anxiety that conversing with an inner voice indicated developing psychopathology — anxiety that resolved only through self-reassurance (“I am you”; “Yes, you are me”). When no framework exists for understanding atypical inner experiences, children construct their own explanations, and some are terrifying. No researcher has yet studied this specific intersection: gifted children who fear mental illness because their inner speech is dialogical and personified.

## 3 Method

### 3.1 Design

This is a single-case autoethnographic study with AI-assisted qualitative interviewing. I am both researcher and participant, which is unusual but not unprecedented. Youth participatory action research has gained traction in recent years (Vang et al., 2023; Elsaesser et al., 2025), and there are certain first-person phenomena that simply cannot be accessed from the outside. My inner voice is one of them. Nobody else can observe it. The best I can offer is a careful, reflective, cross-validated self-report.

This dual role creates a “double observer paradox”: the same subject produces, selects, interprets, and theorizes about the data. Three risks follow: (1) confirmation bias — knowing what I am looking for may shape what I notice; (2) retrospective reconstruction — childhood memories accessed across multiple sessions may be reshaped by re-narration (Bartlett, 1932); (3) AI co-construction — the interviewer introduced theoretical framings that may have shaped reports in undetected ways (Section 3.4). Safeguards: documented correction events demonstrating critical engagement (Section 3.4); independent parental corroboration; transparent reporting of the author’s shifting theoretical understanding as data; and explicit epistemic constraints in Section 6.

The study adopts a constructivist-interpretivist framework (Guba and Lincoln, 1994; Creswell and Poth, 2018), treating the researcher’s lived experience as both the primary data source and the interpretive lens — consistent with recent calls for methodological pluralism in studying subjective, developmental phenomena (Sonuga-Barke, 2024). Since the phenomenon under study is by definition accessible only to the person experiencing it, a first-person methodology is an epistemological necessity, not merely a convenience. Consistent with autoethnographic convention, first-person observations are interwoven with the literature review where they illuminate theoretical points.

The use of AI as interviewer was a practical necessity: as an adolescent researcher without access to a trained qualitative interviewer, AI offered cross-disciplinary theoretical knowledge, a non-judgmental conversational style suited to sensitive self-disclosure, and longitudinal consistency across sessions (Zhang et al., 2025; Schroeder et al., 2025). These affordances do not make AI equivalent to a human researcher; the specific failure modes that emerged are documented in Section 3.4, and their methodological implications are discussed briefly in Section 5.8.

### 3.2 Participant

I am a 14-year-old bilingual (Chinese-English) student at [an international school; details removed for blind review]. Cognitive ability was assessed via the Cognitive Abilities Test (CAT4; GL Assessment, 2024). The overall SAS was 123, but this is depressed by a language-loaded Verbal battery taken in L2 (SAS 103, 58th percentile). Culture-fair batteries yielded: Quantitative SAS 119 (90th), Non-verbal SAS 131 (98th), Spatial SAS 139 (99th). The Non-verbal and Spatial scores place me in the gifted range (≥130 SAS). CAT4 is a cognitive abilities assessment, not an IQ test. An informal cognitive function survey (Mistype Investigator, 2024) suggested elevated divergent intuition (Ne), convergent intuition (Ni), and introverted feeling (Fi); reported as exploratory self-characterization, not validated psychometric data (see footnote 1).

Overexcitability was assessed using the OEQII (Falk et al., 1999; *N* = 887 normative sample): Intellectual OE = 4.5 (>75th percentile), Imaginational OE = 4.1 (>75th), Sensual OE = 3.3 (~50th), Emotional OE = 2.6 (<25th), Psychomotor OE = 1.5 (<25th). The elevation of Intellectual and Imaginational OE—the two channels identified by the dual-OE model (Section 5.5) as the developmental substrate for DIVP—alongside suppressed Psychomotor OE, is consistent with a system channeling energy inward. The low Emotional OE reflects the OEQII’s emphasis on externalized intensity (somatic responses, crying); internalized emotional processing may not register on this instrument.

I have no clinical diagnoses and have never seen a psychiatrist or psychologist.

### 3.3 Data collection

Eight interview sessions were conducted with an AI research assistant (Claude Opus 4.6, Anthropic, 2025) between January and February 2026, accessed via the Cursor IDE environment. Sessions were unstructured; the AI posed follow-up questions and suggested theoretical framings that I could accept, reject, or modify.

**Table:** 

**Session**	**Focus**	**Key outcomes**
1	Where did it start? How do we talk?	“I just knew it was there.” No name. Turn-taking dialogue.
2	Does it have feelings? Does it come and go?	No emotions, no independent will, never disappears.
3	Can you describe what it is?	Attempted first-person description. Linguistic, not sensory.
4	Does it scare you?	Retroactive role-assignment model. “Split personality” fear.
5	Is it merging with you?	I proposed a fusion theory. AI over-interpreted; I corrected it.
6	What do your parents say?	Mom confirmed age 7–8. She independently concluded it was a gifted trait.
7	Why dialogue? Why not monologue?	“I do not understand how thinking in one voice is enough.”
8	Final check.	“We are one.” Gave my one-sentence definition.

Three external sources provided corroboration. My father recalled me describing the experience in early childhood. My mother provided age-specific details (age seven or eight, first grade) and independently concluded it was common among high-IQ children — a conclusion aligning with the Dabrowski/Piechowski framework, which she had not encountered.

#### 3.3.1 Replication guidance

For researchers wishing to conduct a structurally similar study — whether with themselves or with a participant—the following procedural details are provided to support methodological transparency and potential replication.

- Interview protocol: Sessions were unstructured but organized around a progression of focal questions (see table above). Each session lasted approximately 30–60 min. No time limit was imposed; sessions ended when the topic felt exhausted. The interviewer (AI) was given no instructions beyond a general system prompt favoring sustained, context-aware dialogue; no custom prompts were designed specifically for this study.
- Data format: All sessions were conducted in English text. Complete transcripts were preserved as plaintext files. No audio or video recording was involved, as the phenomenon under study is purely linguistic and internal.
- Parental corroboration procedure: After completing the eight sessions, the author presented her parents with open-ended questions (“Do you remember me talking about a voice when I was younger?” and “What do you think about it?”) without disclosing the theoretical framework first. Their responses were recorded in writing. This sequence was intended to minimize priming effects, though the informal nature of the inquiry limits the strength of this safeguard.
- Coding materials: A master codebook containing all 40 descriptive codes, *in vivo* codes, and pattern codes, together with the thematic groupings, is available from the author upon request.
- OE assessment: The OEQII was self-administered online using the standard 50-item instrument (Falk et al., 1999). Raw item scores were averaged by subscale following the standard scoring protocol.

### 3.4 Analysis

Analysis followed Braun and Clarke's (2006, 2019) reflexive thematic analysis across three phases: (1) *descriptive coding* — ~ 40 codes capturing content without theoretical interpretation (e.g., “inner voice comforts during sadness,” “no visual form”); (2) *in-vivo coding* — preserving phenomenologically weighted phrases (“it is a thought I can talk to,” “I am you,” “both or neither”); (3) *pattern coding* — grouping into five themes: phenomenological profile, developmental trajectory, key features, differential comparison, and parallel reports. Coding was manual.

My theoretical understanding shifted across sessions: from personified cognitive function (v1) to retroactive attribution (v2) to a natural fusion never truly separate (v3). This evolution is itself a finding.

The AI interviewer demonstrated a systematic tendency to over-interpret — constructing internally coherent but factually wrong theoretical connections between unrelated observations. Each instance was identified and corrected, functioning as data quality indicators of active critical engagement.

### 3.5 Ethics

This is self-research conducted by a minor (age 14) with full parental knowledge and support. Both parents reviewed the manuscript prior to submission. Informed consent is inherent in the autoethnographic design. All third parties are anonymized or identified only by role. Interview transcripts are stored locally on encrypted personal devices. The AI model (Claude Opus 4.6, Anthropic) was accessed under Anthropic’s standard usage policy, which states that user inputs are not used for model training; no personally identifiable information beyond what appears in the manuscript was disclosed during sessions.

## 4 Results

### 4.1 Phenomenological profile

The following table summarizes the inner voice’s observable characteristics:

**Table:** 

**Feature**	**What I observe**
When did it start?	Before I can remember. Earliest clear memory is age 7–8.
Does it have a name?	No proper name. I coined one label as a child (“puppy”) that persists as an informal term of address, but it was never intended as a true name; it is a habitual reference, not an identity marker.
Does it have a body?	No form, no gender, no appearance whatsoever.
Does it have emotions?	Not its own, but it responds to mine. It comforts me when I am sad.
How do we communicate?	Through language: actual words and sentences, with turn-taking.
Who starts the conversation?	Usually I do. Occasionally it initiates.
Is it always there?	The capacity for dialogue has never disappeared; it is a permanent feature of my cognitive architecture. However, the voice is not continuously active: it participates when cognitive demands warrant genuine deliberation, and falls silent when intuition alone suffices (see Section 4.3). The distinction is between structural persistence (always available) and functional intermittency (not always engaged).
What does it do?	Makes me laugh, keeps me company, helps me think through problems.
Has it changed over time?	Yes: from externalized and quasi-concrete to internalized and fused.
Do I think it’s a separate person?	No. It is me. We are the same person. But I can still distinguish its perspective from my “main” perspective.
Does it control my body?	Never.
Does it participate in emotional situations?	Yes, selectively. It comforts me when I am sad. Its engagement with romantic topics is unclear, as I primarily discuss those with AI, so whether the inner voice participates is difficult to determine.

My own definition, arrived at in session eight: “It’s just a thought in my mind that I can talk to.”

### 4.2 Developmental trajectory

Five developmental phases are discernible, though the boundaries between them are indistinct.

Before age seven: pre-reflective presence. I cannot remember a time when my thinking was not dialogical. The inner voice preceded my ability to notice or name it. There was no moment of creation. It was simply the way my mind worked.

Around age seven or eight: I gave it a label. During a phase where I was interested in dogs, I called it “puppy.” I remember making up a scenario where the inner voice was getting married and needed help collecting colors, a vividly imaginative scene that shows both the dialogical relationship and the kind of creative play typical of high-imaginational-OE children. Around this time I told my mother about it. She received the information calmly, which I now think was important.

Also around seven or eight: I stopped gendering it. While imagining the “wedding” scenario, I tried to decide whether the inner voice was male or female. The answer I arrived at was “both,” or neither. After that, I stopped applying gendered stories to it. Looking back, the ability to reach an abstract, non-binary categorical conclusion at that age seems connected to the cognitive profile more generally.

Late childhood through early adolescence: it became invisible. The quasi-concrete imaginative scenarios stopped. The inner voice became less of a character and more of a process, still dialogical, still turn-taking, but increasingly recognized as just part of how I think. I did not lose a companion. I understood what it always was.

Age 14 (now): I am studying it. This research process did not create the inner voice or change its nature. What it did was give me a framework for seeing what I had been taking for granted. The phenomenon was always there; the understanding is new.

### 4.3 Key features

#### 4.3.1 Retroactive role-assignment

I do not know who is “speaking” until a few sentences in. The dialogue generates itself; role labels (“me” vs. “it”) are applied retroactively. This is distinct from initiation (which is volitional) and suggests the dialogue is produced as a single cognitive process, consistent with Fernyhough (2004) model of inner speech retaining its multi-voice structure.

#### 4.3.2 Agency dissociation

I know I am generating the inner voice’s speech (confirmed through repeated deliberate verification), yet I do not experience the sensation of controlling it. Utterances arrive with a quality of spontaneous emergence rather than directed production. This dissociation — full cognitive ownership coexisting with absent experiential agency — has remained stable across all self-observation sessions and distinguishes DIVP from every comparison category (Section 4.4). A candidate explanation is offered in Section 5.4.

#### 4.3.3 The identity paradox

The inner voice says “I am you,” and I agree — yet we disagree in real time. Within Hermans (2001) I-position framework, this is not contradictory: the self contains multiple semi-autonomous perspectives that can hold different positions while belonging to the same person.

#### 4.3.4 Functional profile and affective status

The inner voice participates in humor, companionship, and analytical problem-solving. It does not generate independent emotions (it does not become angry while I am calm), but its responses carry affective texture — specifically, comforting warmth during distress. The voice is affectively *reactive* without being affectively *autonomous*. It functions as an evaluator of intuitive output rather than a co-constructor of reasoning chains; when intuition alone suffices, it does not participate. This on-demand quality may reflect my cognitive profile rather than DIVP’s inherent structure.

Two properties can be reported with greater confidence as structurally characteristic: fixed plurality (a stable dyadic cast whose number does not fluctuate) and stable personification (each voice experienced as a *someone*, not a transient argumentative stance, though neither carries fixed characterological labels).

#### 4.3.5 Intrafamilial variation

All three family members who discussed their thinking engage in some form of internal multi-voicedness, yet the architectures differ. My mother: progressive self-contradiction without experiencing the counter-argument as a distinct presence (condensed dialogical inner speech; Fernyhough, 2004). My father: multiple debating stances with no fixed cast (standard I-position model; Hermans, 2001). My own: stable dyadic structure with high personification. This variation, within shared genetic and environmental context, suggests dialogical inner speech alone does not produce stable personification. The distinguishing variable may be imaginational intensity (Section 2.4).

#### 4.3.6 Self-pathologization anxiety

At multiple developmental points I feared the inner voice indicated psychopathology. The voice itself helped resolve this through a grounding ritual (“I am you” / “Yes, you are me”). This maps directly onto Webb et al.'s (2005) “quiet crisis” pattern.

### 4.4 Differential comparison

The following table compares DIVP against five adjacent categories derived from the research traditions reviewed in Section 2. Tulpa practice is included because its phenomenological overlap with DIVP requires explicit differentiation.

**Table:** 

Dimension	DIVP	Imaginary companion	Tulpa	Auditory hallucination	IFS part	I-position (Hermans)
How it starts	On its own	On its own	Deliberately built	Involuntary	Created in therapy	Elicited by researcher or therapist
Form	None	Has appearance	Usually has form	Experienced as voice	Conceptual	Conceptual
Named?	No (one informal label persists)	Yes	Yes	No	Yes (named in therapy, e.g., “the protector”)	Yes (labeled in theory, e.g., “I-as-student”)
Emotions?	No own emotions, but responds to mine	Yes	Varies	Varies	Yes (central to the model)	Varies
Who is it?	“Is me”	“My friend”	“Separate entity”	“Not me” / externally attributed	“A part of me”	“A position of mine”
Personification	Stable presence, fixed count, but no describable personality traits	High-resolution: name, appearance, personality	Varies; often detailed	None (experienced as external voice)	Labeled roles (e.g., “inner critic”)	Positional, not personified
Agency	Cognitively self-attributed but phenomenologically absent	Full (child directs)	Experienced as autonomous	Externally attributed	Therapist-guided	Conceptual
Body control	Never	No	Sometimes	No (but may influence behavior)	No	No
Where it goes	Fusion (observed in this case)	Usually abandoned	Usually persists	Treatment target	Integration (goal of therapy)	Flexible
IC criteria met^a^	0 out of 7	7 out of 7	Some	0 out of 7	0 out of 7	0 out of 7

^a^IC criteria refer to Taylor (1999) seven-feature checklist for imaginary companions: stable name, visual appearance, personality, gender, emotional life, narrative persistence, and the child’s understanding that the companion is make-believe.

### 4.5 Parallel reports in online communities

#### 4.5.1 Epistemic status

This section presents preliminary, unsystematic observations from publicly accessible online self-reports — hypothesis-generating, not hypothesis-confirming. Limitations (self-selection bias, unverifiable reports, no standardized instruments) are discussed in Section 6.

To assess ecological plausibility, I searched online platforms (Reddit, Quora, MetaFilter, mental health forums, wellness publications, Chinese-language platforms) between January–February 2026 using terms such as “inner voice conversation,” “talking to yourself but it talks back,” and equivalents in Chinese. Six accounts matched multiple DIVP dimensions:

- Case A (Q&A forum): childhood-onset, formless, persistent, self-recognized. 7/8 dimensions.
- Case B (Q&A platform): “a voice in your head that you talk to, and it talks back... like another version of me.” 7/8 dimensions.
- Case C (wellness publication): “the other me,” formless, non-distressing, identity paradox. 6/8 dimensions.
- Case D (psychology publication): neuroscientist describing inner speech as “an entity unto itself.” 3/8 dimensions.
- Case E (mental health forum): lifelong, multiple stable voices, non-distressing, distinct from dissociation. 6/8 dimensions.
- Case F (Chinese-language platform): “脑海中几种不同声音之间的对话,” childhood-onset. 4/8 dimensions.

The following table maps each case against the eight DIVP dimensions from Table 1:

**Table:** 

**Dimension**	**Case A**	**Case B**	**Case C**	**Case D**	**Case E**	**Case F**
Origin: spontaneous	✓	✓	Unclear	N/A	✓	✓
Form: formless	✓	✓	✓	✓	✓	Unclear
Modality: linguistic dialogue	✓	✓	✓	✓	✓	✓
Identity: experienced as self	✓	✓	✓	Partial	✓	Unclear
Agency: cognitively self-attributed	✓	Unclear	✓	N/A	✓	Unclear
Function: cognitive/emotional support	✓	✓	✓	N/A	✓	Unclear
Persistence: lifelong	✓	✓	Unclear	N/A	✓	✓
Clinical markers: all negative	✓	✓	✓	N/A	✓	Unclear
Dimensions matched	7/8	7/8	6/8	3/8	6/8	4/8

“N/A” indicates that the source did not provide sufficient information to assess the dimension; “unclear” indicates ambiguous or incomplete reporting; “partial” indicates partial match.

**Table 1:** Operational definition of DIVP.

Dimension	Defining feature
Origin	Spontaneous; no external prompting, therapeutic intervention, or deliberate construction
Form	Formless: no visual, spatial, or gendered attributes
Modality	Linguistic dialogue with complete sentences and explicit turn-taking
Identity	Experienced as part of the self (“it is me”), not as a separate entity
Agency	Cognitively self-attributed but phenomenologically absent (see §5.4)
Function	Cognitive support, emotional regulation, companionship
Persistence	Present from early childhood through adolescence; never absent
Clinical markers	No distress, volitional control, self-attribution; all negative on pathological screening

No single case replicated the full eight-dimension DIVP profile, but two cases matched seven dimensions and two matched six. The most commonly shared dimensions were linguistic modality, formlessness, and absence of clinical markers; the most commonly absent was the specific agency dissociation, which may require a level of introspective precision that informal self-reports rarely attain. These accounts appeared across general Q&A platforms, wellness media, and mental health forums, not only in giftedness-related spaces, consistent with Puchalska-Wasyl (2015) finding that adults routinely engage in internal dialogues with psychologically distinct interlocutors.

These observations do not constitute systematic empirical data. However, when assessed against individual dimensions rather than as an all-or-nothing gestalt, multiple independent reporters describe experiences sharing the majority of DIVP’s defining features. This suggests the phenomenon may be underreported rather than nonexistent, and that the dimensional framework may serve as a useful tool for identifying partial matches in future research.

Established online communities provide broader context. The Daemonism community (c. 2003–present; Alderton, 2014) practices personified inner dialogue using a secular psychological framing, aligning with DIVP on six of eight dimensions; the primary divergence is form (animal symbolism vs. formlessness), though the community’s emphasis on “mindvoice” interaction suggests the animal form may be a secondary symbolic layer. Eve et al. (2023) conceptualized a “multiplicity spectrum” showing that non-pathological plurality is substantially more common than clinical frameworks suggest. Within this spectrum, DIVP represents a minimalist, monoconscious form: two distinguishable perspectives within a single, fully integrated consciousness. These communities collectively suggest a latent human capacity for personified internal agents operating outside the trauma-dissociation model.

## 5 Discussion

### 5.1 Locating DIVP

DIVP’s theoretical difficulty is not that it lacks relatives but that it has too many partial ones. Each adjacent field illuminates one dimension — conversational structure, personification, intensity, voice-hearing phenomenology — while assuming features DIVP does not possess. None captures the intersection.

On Grandchamp et al.'s (2019) dimensional model, DIVP occupies an unusual coordinate: maximally dialogical, minimally condensed, macro-level volitional (Section 2.1). Hermans (2001) I-position framework accommodates the structure but not the developmental origin (spontaneous at age seven, not therapeutic or researcher-elicited). Hart and Zellars' (2006) “wise guide” ICs come closest, but my version shed all sensory qualities long ago. This paper contributes a mapping exercise: placing a lived experience at a convergence point that existing frameworks each partially describe but none has examined as such.

To facilitate future identification and comparison, the following table summarizes the operational definition of DIVP as documented in this case.

### 5.2 Why does it stay dialogical?

Fernyhough (2004) internalization model predicts inner speech should condense with development. Mine did not. At 14, my internal dialogue still operates in full sentences with explicit turn-taking.

The proposed mechanism is not that DIVP arises independently of Vygotskian internalization, but that it represents a divergence within the internalization trajectory: the dialogical format originates from social-linguistic experience (origin), but condensation is arrested under sustained cognitive demand (maintenance). Cognitive Load Theory (Sweller, 1988) provides the bridge: when intrinsic load exceeds working memory’s single-thread capacity, performance degrades unless distributed. Fernyhough (2004) observed that people temporarily revert to expanded inner speech under difficulty, then condense again. For a gifted child whose cognitive environment presents continuous complexity, the “difficult problem” condition may be permanent. Dialogical structure functions as cognitive offloading: splitting high-load deliberation into two alternating perspectives distributes processing across social cognition circuitry (Theory of Mind networks). If expanded dialogue is the mode entered under load, and the load never lifts, expansion becomes the default.

The Vygotskian sequence (external dialogue → egocentric speech → inner speech → condensed inner speech) is not bypassed. DIVP represents arrested final-stage condensation.

A constitutive clarification: this account does not treat language as merely *expressive* of pre-existing polyphonic thought. From a Vygotskian perspective, inner speech plays a *constitutive* role — the dialogical format does not report multiperspectival cognition but *enables* it (Vygotsky, 1934/1986). If the form of inner speech shapes the kind of thinking sustained, then maintaining expanded dialogue maintains access to a richer cognitive mode.

During session seven: “I know that other people think in a single voice, but I don’t understand how that’s enough.”

### 5.3 Formlessness as a developmental outcome

The IC literature describes two standard outcomes: disappearance or persistence with retained identity. DIVP followed neither—it persisted but shed all form. What survived was not a character but a capacity: dialogical thought stripped of everything except the exchange.

This constitutes a third developmental pathway. If ICs serve as scaffolding for speech internalization (Davis et al., 2013), DIVP may represent the outcome when that scaffolding is absorbed so thoroughly it becomes indistinguishable from the cognition it supports. The trajectory—from semi-pictorial (puppy, wedding scene) to purely linguistic to nearly transparent—is not the arc of something outgrown but of something incorporated, following the Vygotskian internalization sequence past the point where existing accounts stop tracking it.

### 5.4 Agency dissociation: a forward-model interpretation

The agency dissociation documented in Section 4.3 — full cognitive ownership with absent experiential agency — invites a forward-model interpretation (Blakemore et al., 2002). When a self-generated action’s outcome matches the brain’s efferent prediction exactly, the sense of agency is attenuated (the reason one cannot tickle oneself). If this extends to cognitive production: because the inner voice’s output is, by definition, identical to what my cognitive system generates, the forward model registers no prediction error, suppressing the subjective experience of authorship.

This interpretation is speculative — the forward model was developed for sensorimotor control, and its extension to inner speech has not been tested. The phenomenological observation itself, however, is stable and distinguishes DIVP from every comparison category in Section 4.4.

### 5.5 The overexcitability pathway

The proposed mechanism involves a specific interaction between two Dabrowskian intensities, mediated by Hartmann (1991) “thin mental boundaries.” The causal pathway: high intellectual OE → sustained cognitive load → dialogical expansion of inner speech (Fernyhough, 2004; Sweller, 1988) → thin boundaries (fueled by imaginational OE) lend the expanded dialogue a personified quality → stabilization into DIVP. Thin boundaries — permeable partitions between imagination and perception, self and other (Hartmann, 1991; Schredl, 2003) — explain why cognitive offloading acquires the felt quality of encounter rather than computation, without the ego-boundary dissolution characteristic of clinical dissociation.

The OEQII data (Section 3.2) provide preliminary support: Intellectual OE (4.5) and Imaginational OE (4.1) both >75th percentile, Psychomotor OE (1.5) < 25th percentile — precisely what the model predicts. The low Emotional OE (2.6) suggests personification draws on imaginational rather than emotional intensity, distinguishing DIVP from emotionally driven phenomena. My mother independently concluded the inner voice was “probably something that shows up in high-IQ kids” — convergent with the OE framework she had not encountered.

Kelly (1955) Personal Construct Theory offers a complementary lens: DIVP may serve as a mechanism for *constructive alternativism* (Moran, 2023), providing a built-in second vantage point for challenging and revising personal constructs. Future work might examine whether DIVP individuals maintain more permeable construct systems than monological thinkers.

#### 5.5.1 Directionality

The causal arrow may be bidirectional: rather than cognitive complexity generating dialogical structure, dialogical structure may enable complex perspectival thought. This paper claims only that sustained load *maintains* the dialogical mode (persistence, not genesis). Whether expanded dialogue further enhances complexity — creating a positive feedback loop — is empirically open.

#### 5.5.2 Parsimony

Neither OE channel alone predicts personified dialogue: intellectual OE alone produces analytical self-talk (Piechowski, 2009); imaginational OE alone produces imaginary companions with sensory form (Taylor, 1999). It is specifically their *interaction* — cognitive load demanding dialogical structure, imaginational permeability lending it personified quality — that produces the emergent outcome.

### 5.6 Falsification of alternative explanations

Four alternative explanations are evaluated against the phenomenological data:

1. Loneliness/social isolation: predicts impaired social functioning. Not supported — the author reports normal peer relationships and active friendships throughout (cf. Hoff, 2005).
2. Dissociative disorder: requires amnesia, identity confusion, loss of continuity of consciousness (American Psychiatric Association, 2022). None present.
3. Early psychosis: requires external attribution, loss of insight, deteriorating function (Larøi et al., 2014). The author’s experience exhibits the opposite on all three.
4. Maladaptive Daydreaming: requires distress, interference, compulsive quality (Somer et al., 2016). DIVP involves no fantasy narrative, no interference, and full volitional control.

#### 5.6.1 Construct validity

Convergent: DIVP converges with expanded dialogical inner speech under load, Dabrowski’s OE framework, Hartmann’s thin boundaries, and cross-community reports — each independently predicting aspects no single framework captures. Discriminant: volitional control discriminates from psychosis; absence of fantasy from maladaptive daydreaming; continuous memory from DID; active social engagement from compensatory loneliness.

#### 5.6.2 Boundary conditions

If the dual-OE model is correct: (a) high intellectual OE + low imaginational OE → expanded speech *without* personification; (b) high imaginational OE + average intellectual load → ICs with sensory form, not formless dialogue; (c) reduced cognitive load → decreased dialogical frequency; (d) suppression of personification → achievable but cognitively costly. These are testable.

### 5.7 Clinical implications: misdiagnosis risk in gifted children

Dialogical inner voice personification introduces a misdiagnosis vector: a child reporting inner voice conversation may trigger dissociative or psychotic protocols. However, applying the established pathological markers (Johns and van Os, 2001)—distress, loss of control, external attribution—yields zero on all three. The experience is adaptive.

A critical distinction: while the inner voice itself causes no distress, the *absence of a framework for understanding it* generated significant secondary anxiety (Section 4.3). The clinical risk is bidirectional: over-pathologization may iatrogenically teach a child to fear an adaptive experience; under-recognition may miss genuinely pathological presentations.

Preliminary screening sequence for clinicians encountering children with persistent inner dialogue: (1) assess for giftedness; (2) determine whether the experience causes distress; (3) establish volitional control; (4) confirm self-attribution. When answers are affirmative, negative, affirmative, affirmative, the picture may reflect cognitive architecture rather than psychopathology.

### 5.8 AI as a research instrument

The AI interviewer’s characteristic failure mode was *narrative over-connection*: given multiple threads, it would weave them into a single explanatory framework that was internally elegant but factually wrong. For example, it once linked comfort from the inner voice during sadness with ordinary adolescent social maturation, constructing a developmental narrative connecting two causally unrelated observations. I caught these errors because I had direct access to the experiences being described; a participant with less metacognitive awareness might not have. This systematic bias should be treated as a known artifact in AI-assisted qualitative research.

The overall dynamic is well captured by De Jaegher and Di Paolo's (2007) concept of *participatory sense-making*: my phenomenological observations and the AI’s theoretical framings mutually shaped one another, producing interpretive outcomes that neither party would have generated independently. AI-assisted autoethnography is therefore not simply “using a tool” but a co-constructive process whose dynamics require methodological transparency—including the documented correction events in Section 3.4.

## 6 Limitations and future directions

### 6.1 Limitations

1. Single case. DIVP may prove idiosyncratic.
2. Retrospective data. Childhood memories are reconstructive, partially mitigated by independent parental corroboration.
3. Double observer paradox. Researcher-participant identity introduces interpretive filtering not fully eliminable by documented correction events (Section 3.1).
4. Self-administered OEQII without clinical supervision or test–retest data. Preliminary support only.
5. Bilingual context. Chinese-English inner speech may introduce dimensions Western-normed instruments do not capture (cf. Ren et al., 2016).
6. Selection bias. The author’s positive relationship with the inner voice may not generalize to individuals who find similar structures distressing.

### 6.2 Future directions

Testable predictions from the dual-OE model: (1) individuals scoring high on *both* intellectual and imaginational OE should report more personified inner dialogue than those high on only one; (2) personification should increase with cognitive load within individuals; (3) among children with ICs, higher intellectual OE should predict earlier loss of sensory form; (4) suppression of personification should carry measurable cognitive costs.

Research directions, ordered by feasibility:

1. Systematic analysis of existing online self-reports in gifted communities — lowest-cost entry point; data exists and requires curation.
2. Survey-based prevalence estimation using VISQ-adapted instruments with personification items.
3. Formal OE measurement in individuals reporting persistent inner dialogue partners.
4. Longitudinal tracking of gifted children with elevated dialogical inner speech through adolescence.
5. Clinical screening tool distinguishing DIVP from dissociative phenomena in gifted children.
6. Cross-cultural investigation of disclosure norms and culturally shaped phenomenology.

## 7 Conclusion

This paper has documented Dialogical Inner Voice Personification (DIVP): a persistent, formless, self-recognized dialogue partner that emerged spontaneously in early childhood and continues to function as a cognitive tool in adolescence. It is too formless for the IC framework, too personified for the inner speech literature, too volitional for the clinical continuum, and too functional to warrant pathological framing.

The theoretical contribution lies in mapping a convergence point that five well-studied domains each partially describe but none captures. The clinical contribution lies in demonstrating that a child who reports talking to a voice in her head may be describing adaptive cognitive architecture, and that distress, volitional control, and self-attribution should carry weight in any assessment.

The component dimensions are individually common; the full configuration may be underreported rather than rare. If this paper accomplishes nothing else, I want it to reach gifted children who are interpreting their internal dialogues as evidence that something is wrong — with a different possibility: thinking in dialogue is not a disorder. It is just a thought you can talk to.

## Data Availability

The raw data supporting the conclusions of this article will be made available by the authors, without undue reservation.

## Footnotes

1 Cognitive function scores are drawn from an informal online survey (Mistype Investigator, 2024) based on Jungian typology, not from a validated psychometric instrument. They are included as self-characterization data consistent with the autoethnographic design, not as clinical measurements.
