# Supplementary material for: “A thought I can talk to”: dialogical self-regulation through spontaneous inner voice personification in a gifted adolescent
Source: Front Psychol. 2026 May 19;17:1815412. doi: 10.3389/fpsyg.2026.1815412 (PMC13226489; doi:10.3389/fpsyg.2026.1815412)
Supplement: Supplementary file 1 [file Data_Sheet_1.PDF]

OEQII Score Report

Participant: Author of "A Thought I Can Talk To: Dialogical Inner Voice Phenomena in a Gifted Adolescent" **Instrument:** Overexcitability Questionnaire–Two (OEQII; Falk, Lind, Miller, Piechowski, & Silverman, 1999) **Date Administered:** February 21, 2026 **Method:** 50-item Likert scale (1 = Not at all like me, 5 = Very much like me), administered via interactive AI-assisted questionnaire with items translated from English to Mandarin and presented individually.

Summary Scores

OE Dimension	Score (1–5)	Percentile (n = 887)	Level
**Intellectual**	**4.5**	**>75th**	Very High
**Imaginational**	**4.1**	**>75th**	Very High
**Sensual**	3.3	~50th	Average
**Emotional**	2.6	<25th	Low
**Psychomotor**	1.5	<25th	Very Low

Normative data from the combined sample (N = 887) reported in Falk et al. (1999), Table 2.

Scoring Method

Each OE dimension is measured by 10 items. Dimension score = sum of 10 item scores / 10, yielding a 1–5 scale. Two items are reverse-scored (Q38, Q44): score = 6 minus raw response.

Item-to-dimension mapping (per OEQII Manual):

- **Psychomotor:** Q2, Q7, Q10, Q15, Q18, Q21, Q29, Q39, Q42, Q50
- **Sensual:** Q3, Q8, Q13, Q27, Q32, Q37, Q38(R), Q45, Q46, Q48
- **Imaginational:** Q1, Q4, Q14, Q20, Q22, Q24, Q28, Q33, Q34, Q47
- **Intellectual:** Q5, Q12, Q16, Q19, Q23, Q25, Q30, Q36, Q40, Q43
- **Emotional:** Q6, Q9, Q11, Q17, Q26, Q31, Q35, Q41, Q44(R), Q49

Raw Item Responses

Item	Resp.	Item	Resp.	Item	Resp.	Item	Resp.	Item	Resp.
Q1	5	Q11	2	Q21	2	Q31	1	Q41	2
Q2	3	Q12	4	Q22	5	Q32	2	Q42	1
Q3	3	Q13	4	Q23	5	Q33	5	Q43	5
Q4	5	Q14	5	Q24	4	Q34	4	Q44	2*
Q5	5	Q15	1	Q25	4	Q35	4	Q45	3

Q6	3	Q16	4	Q26	1	Q36	4	Q46	4
Q7	2	Q17	3	Q27	4	Q37	4	Q47	5
Q8	3	Q18	1	Q28	1	Q38	3*	Q48	3
Q9	3	Q19	5	Q29	1	Q39	1	Q49	3
Q10	1	Q20	2	Q30	4	Q40	5	Q50	2

* Q38 and Q44 are reverse-scored. Q38: raw = 3, scored = 3. Q44: raw = 2, scored = 4.

Dimension Score Calculations

Psychomotor (1.5): Q2(3) + Q7(2) + Q10(1) + Q15(1) + Q18(1) + Q21(2) + Q29(1) + Q39(1) + Q42(1) + Q50(2) = 15 / 10 = **1.5**

Sensual (3.3): Q3(3) + Q8(3) + Q13(4) + Q27(4) + Q32(2) + Q37(4) + Q38(3) + Q45(3) + Q46(4) + Q48(3) = 33 / 10 = **3.3**

Imaginational (4.1): Q1(5) + Q4(5) + Q14(5) + Q20(2) + Q22(5) + Q24(4) + Q28(1) + Q33(5) + Q34(4) + Q47(5) = 41 / 10 = **4.1**

Intellectual (4.5): Q5(5) + Q12(4) + Q16(4) + Q19(5) + Q23(5) + Q25(4) + Q30(4) + Q36(4) + Q40(5) + Q43(5) = 45 / 10 = **4.5**

Emotional (2.6): Q6(3) + Q9(3) + Q11(2) + Q17(3) + Q26(1) + Q31(1) + Q35(4) + Q41(2) + Q44(4) + Q49(3) = 26 / 10 = **2.6**

Reference

Falk, R. F., Lind, S., Miller, N. B., Piechowski, M. M., & Silverman, L. K. (1999). *The Overexcitability Questionnaire–Two (OEQII): Manual, scoring system, and questionnaire.* Institute for the Study of Advanced Development.
